# Supplementary figures and images for: Mesenchyme-derived IGF2 is a major paracrine regulator of pancreatic growth and function
Source: PLoS Genet. 2020 Oct 15;16(10):e1009069. doi: 10.1371/journal.pgen.1009069 (PMC7678979; doi:10.1371/journal.pgen.1009069)

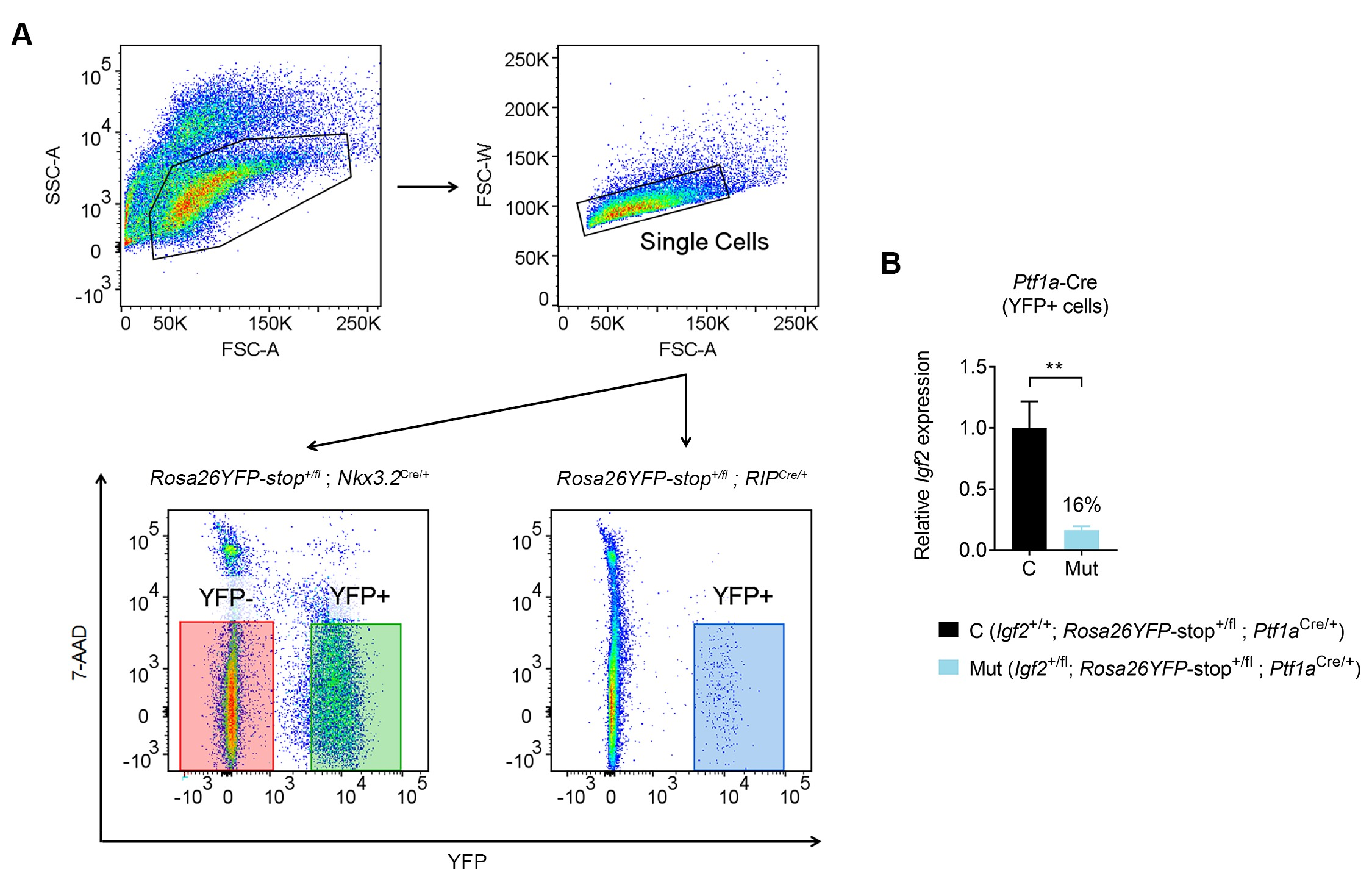

Supplement: S1 Fig — (A) Strategy used for sorting YFP+ and YFP- cells by FACS. Top left panel: side scatter area (SSC-A) versus forward scatter area (FSC-A) plotting was used to identify cells of interest (gated) based on granularity and size, respectively. Top right panel: forward scatter width (FSC-W) versus forward scatter area (FSC-A) was then used to exclude cell doublets. Bottom panels: gates used to discriminate YFP+ (green and blue rectangles) versus YFP- cells (red rectangle), after exclusion of dead cells (positive for 7-Aminoactinomycin D [7-AAD]) in cells isolated from pancreases of two crosses: Rosa26YFP-stop+/fl; Nkx3.2Cre/+ that activates YFP in the mesenchyme-derived cells (bottom left panel) and Rosa26YFP-stop+/fl; RIPCre/+ that activates YFP in the pancreatic beta-cells (bottom right panel). (B) Igf2 mRNA expression measured by qRT-PCR in YFP+ cells collected from offspring with the genotypes indicated. Expression data was normalized to Ppia and shown as averages + SEM relative to levels measured in control (C) littermates, arbitrarily set to 1. Percentage values indicate the level of Igf2 mRNA reduction relative to controls (n = 6 samples/genotype; ** p<0.01 by Mann-Whitney tests). (TIF) [file pgen.1009069.s004.tif]

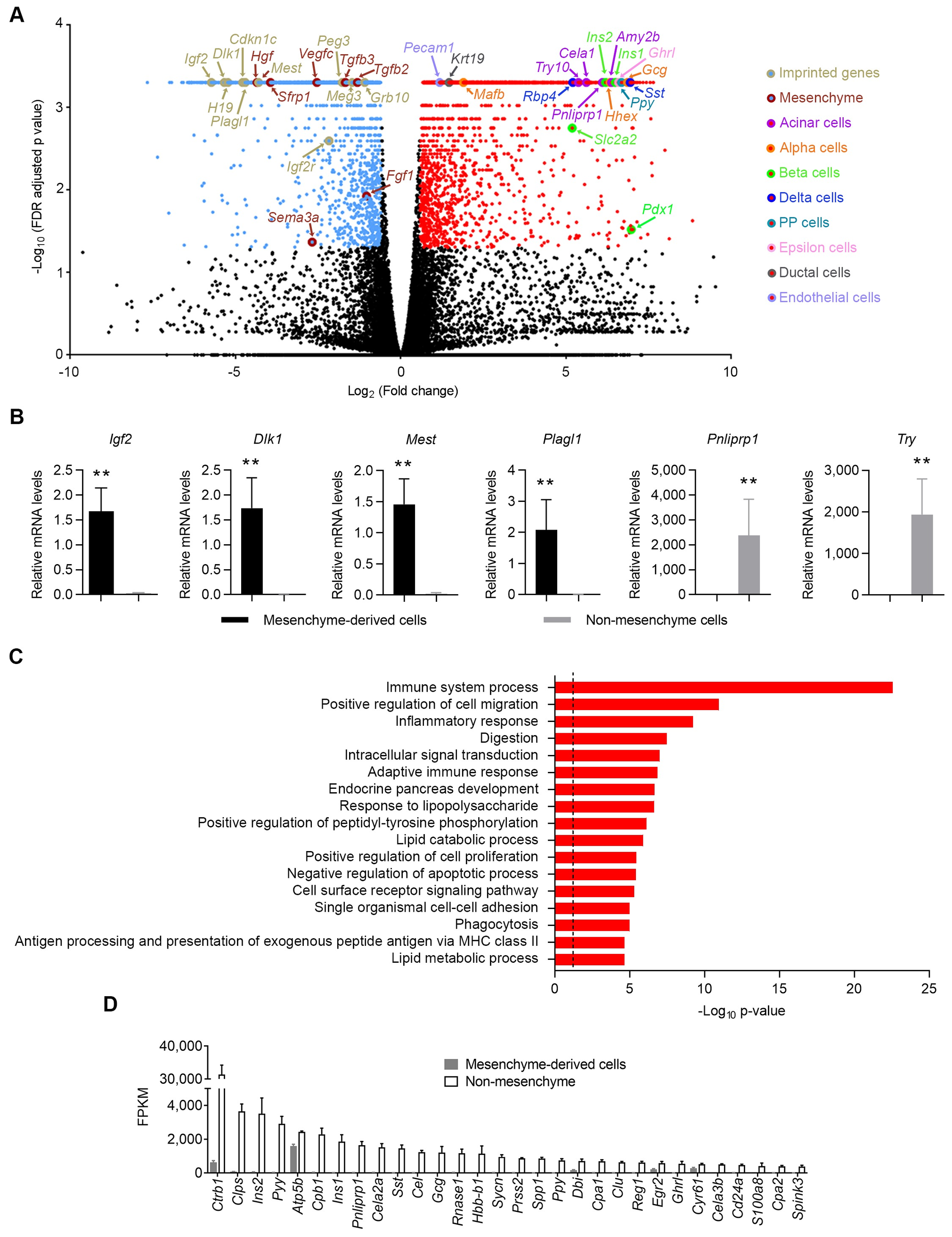

Supplement: S2 Fig — (A) Volcano plot representing all expressed transcripts in pancreatic mesenchyme and non-mesenchyme cells (purified by FACS). For every transcript, the Log2 fold change between mesenchyme and non-mesenchyme cells was plotted against the -Log10 p value (FDR adjusted). Genes significantly enriched, with a fold change >1.5 and FDR adjusted p value <0.05, in mesenchyme are depicted as blue dots (n = 1,902 genes), in non-mesenchyme as red dots (n = 2,212), and those not significantly enriched in either cell fraction are depicted as black dots (all genes are listed in S1 Data). Selected genes of interest, such as known mesenchyme-expressed genes, signature genes for the various non-mesenchyme cell-types and imprinted genes are grouped by colour. (B) Biological validation by qRT-PCR of DEGs between mesenchyme-derived cell and non-mesenchyme cells, identified by RNA-seq (n = 5–6 samples per group). Expression levels were normalized to Ppia. Data is shown are average values; error bars represent SEM; ** p<0.01 by Mann-Whitney tests. (C) Top scoring biological processes containing genes enriched in the pancreatic non-mesenchyme cells as identified by DAVID functional annotation. The dotted line in panel corresponds to a p value of 0.05. (D) Top 30 expressed genes with highest FPKM values in pancreatic non-mesenchyme cells (average FPKM + SEM in mesenchyme and non-mesenchyme cells with n = 4 per group) at P2. Note that all 30 genes are significantly enriched in non-mesenchyme compared to mesenchyme-derived cells (>1.5 fold, FDR adjusted p value <0.05). (TIF) [file pgen.1009069.s005.tif]

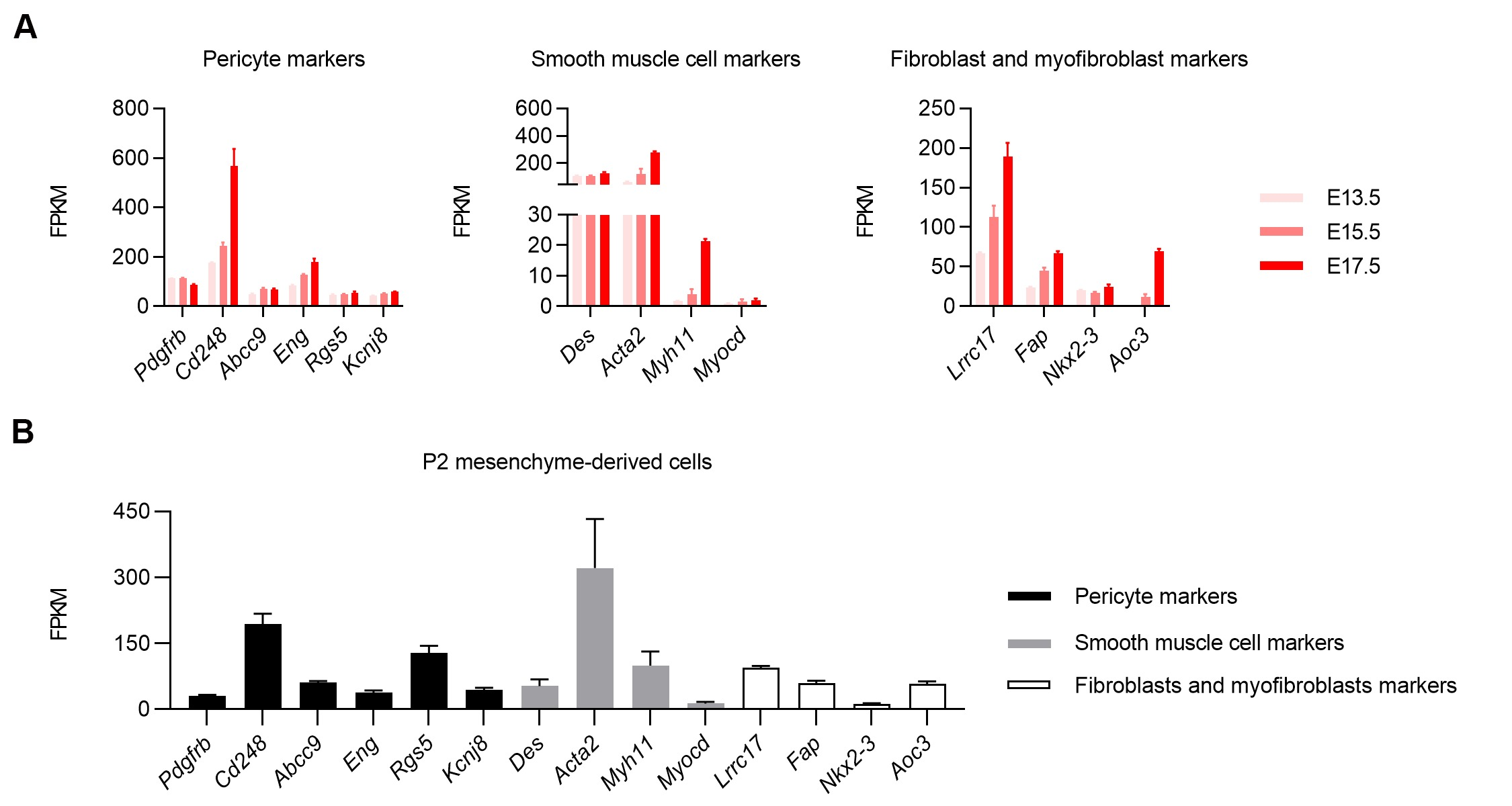

Supplement: S3 Fig — (A) Mesenchyme-derived cells exhibit high expression levels for known markers of pericytes, smooth muscle cells, fibroblasts and myofibroblasts. Expression levels (shown as FPKM + SEM) were calculated by analysing the RNA-seq data obtained in E13.5, E15.5 and E17.5 mesenchyme-derived cells (n = 2 for each developmental time point) by Harari N et al. [3]. (B) Expression levels in P2 mesenchyme-derived cells for the same markers genes shown in panel (A) (n = 4; data shown as FPKM + SEM). (TIF) [file pgen.1009069.s006.tif]

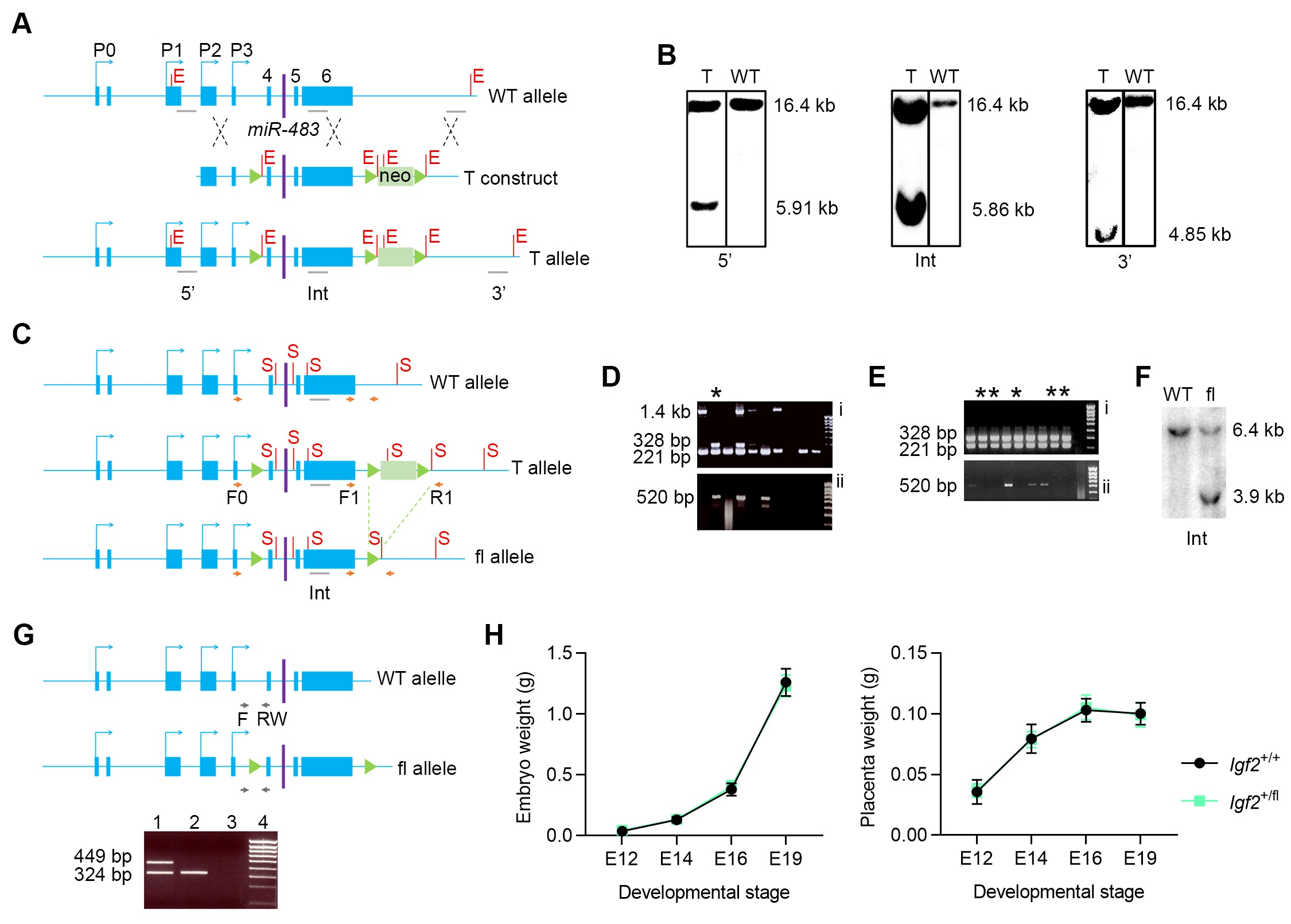

Supplement: S4 Fig — (see also Materials and Methods for further details). (A) Targeting strategy to generate an Igf2 allele with coding exons 4 to 6 flanked by loxP sites (not drawn to scale). Blue boxes–exons; P0-P3 –alternative promoters; green triangles–loxP sites; neo–neomycin cassette; WT–wild-type; T–targeting (construct); E–EcoRI restriction sites; 5’, Int, 3’: location of 5’, internal and 3’ Southern blotting probes, respectively (B) Southern blot confirmation of homologous recombination between targeting vector and endogenous Igf2 sequences in genomic ES cell DNA, digested with EcoRI and hybridized with 5’, Int or 3’ probes. Diagnostic molecular weights (kb) are indicated in each panel. T and WT–targeted and wild-type clones, respectively. (C) Screening strategy for loxP recombination events (not drawn to scale). Correctly targeted ES clones (as shown in (B)) that are transiently exposed to Cre recombinase in vitro will undergo three possible independent recombination events involving the loxP sites, which can be discriminated by PCR and Southern blotting. F0, F1, R1 –PCR screening primers; S–SphI restriction sites; Int–Internal Southern probe (D) Five 96-well plates containing targeted ES cells transfected with Cre recombinase were screened by F1+R1 primer PCR (panel i) or F0+R1 primer PCR (panel ii). F1+R1 PCR products of 221 bp, 1.4 kb and 328 bp are diagnostic of the wild-type allele, neomycin cassette, and neomycin cassette deletion, respectively (panel i). F0+R1 PCR products of 520 bp are diagnostic of a deletion that includes the neomycin cassette and exon4-6 region (in panel (ii)). Since all clones that had deletion of the neomycin cassette (i.e. 328 bp) also had cells with deletion of exon4-6 region (520 bp), the clone indicated with a star in panel (i) was subsequently subcloned for selection of cells with neomycin cassette excision events only. (E) Representative ES subclones (starred) with deletion of the neomycin cassette only (i.e. 328 bp in panel (i) but absence [file pgen.1009069.s007.tif]

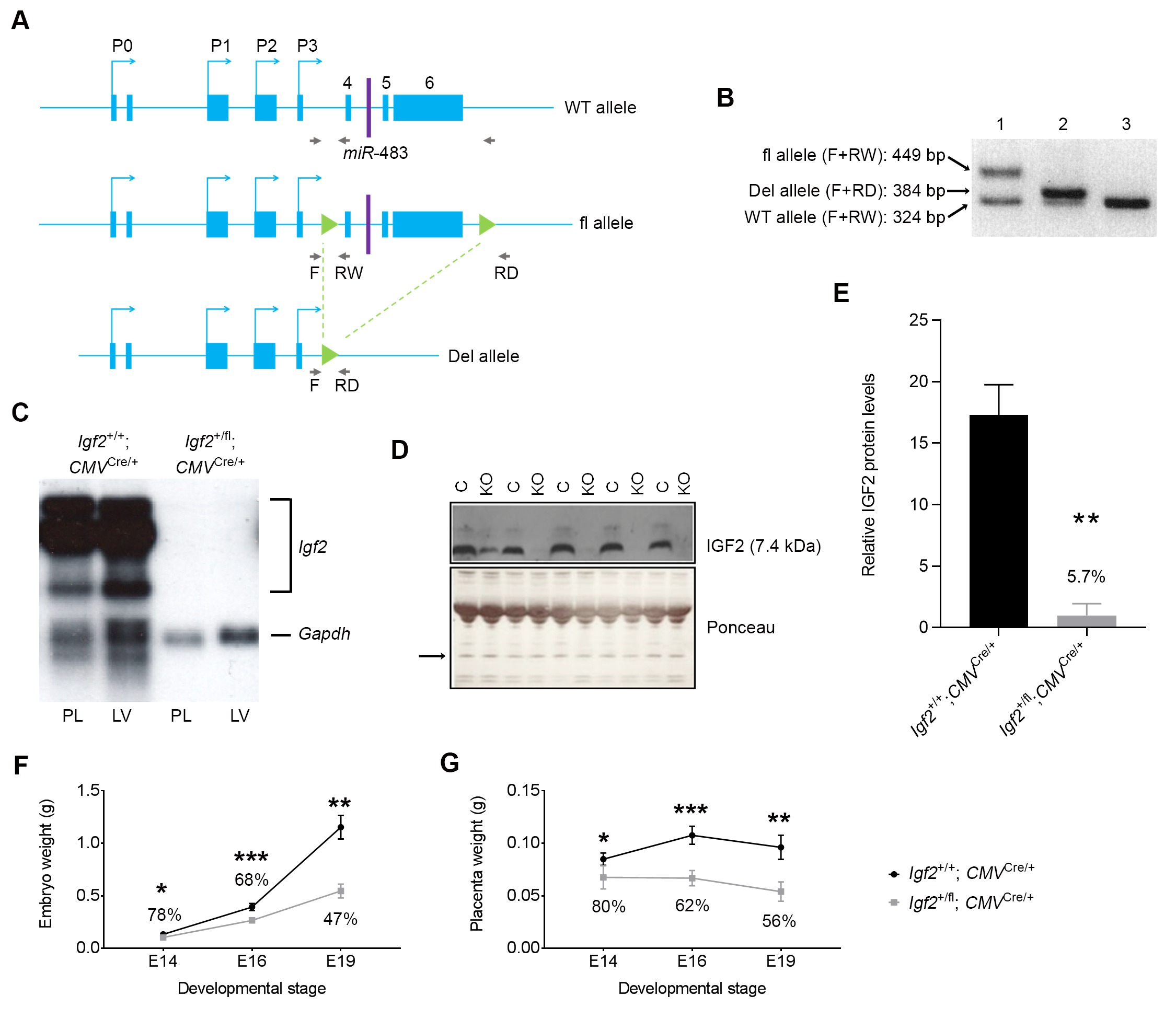

Supplement: S5 Fig — (A) PCR strategy to identify deletion events at DNA level. PCR products obtained in a tri-primer (F+RW+RD) PCR are diagnostic of the wild-type allele (WT: 324bp), floxed allele (fl: 449 bp) and deleted allele (Del: 384bp). Representative tail DNA PCR examples are shown in (B) for Igf2+/fl mice that carry floxed and wild-type alleles (lane 1), Igf2+/fl; CMVCre/+ mice carrying deleted and wild-type alleles (lane 2), Igf2+/+ control littermates with wild-type alleles only. (C) to (G) CMV-Cre mediated deletion of the Igf2 floxed allele in vivo. Heterozygous floxed Igf2 males were mated with females homozygous for CMV-Cre (active in all cells of embryos and placentae), and offspring analysed for levels of Igf2 deletion by northern blotting (C), Western blotting (D) and (E), and growth curves from E14 to E19 of gestation ((F) and (G)). (C) Northern blot analysis of Igf2 mRNA levels, showing wild-type levels of expression in controls (Igf2+/+; CMVCre/+) in both placenta (PL) and liver (LV) at E19, and absence of all Igf2 transcripts upon CMV-Cre mediated deletion of the paternally inherited floxed allele (Igf2+/fl; CMVCre/+). Gapdh–internal control for RNA loading. (D) Western blotting analysis of the mature form of IGF2 in serum samples collected from E19 controls (C: Igf2+/+; CMVCre/+) and mutant (KO: Igf2+/fl; CMVCre/+) embryos. Normalisation of IGF2 expression across samples was performed against Ponceau-stained protein band (arrow), and the relative quantification of IGF2 levels for the two genotypes is shown in (E). Data is shown as average values; error bars represent SEM; ** p<0.01 by Mann-Whitney test. (F) and (G) Mice with a CMV-Cre mediated deletion of the paternally inherited Igf2 floxed allele (Igf2+/fl; CMVCre/+) show a similar embryonic (F) and placenta (G) growth phenotype to Igf2 null mice, i.e. ~ half of the weight of littermate controls (Igf2+/+; CMVCre/+) at the end of gestation (E14: n = 3 litters; E16: n = 9 litters; E19: n = 4 litters). Data is shown [file pgen.1009069.s008.tif]

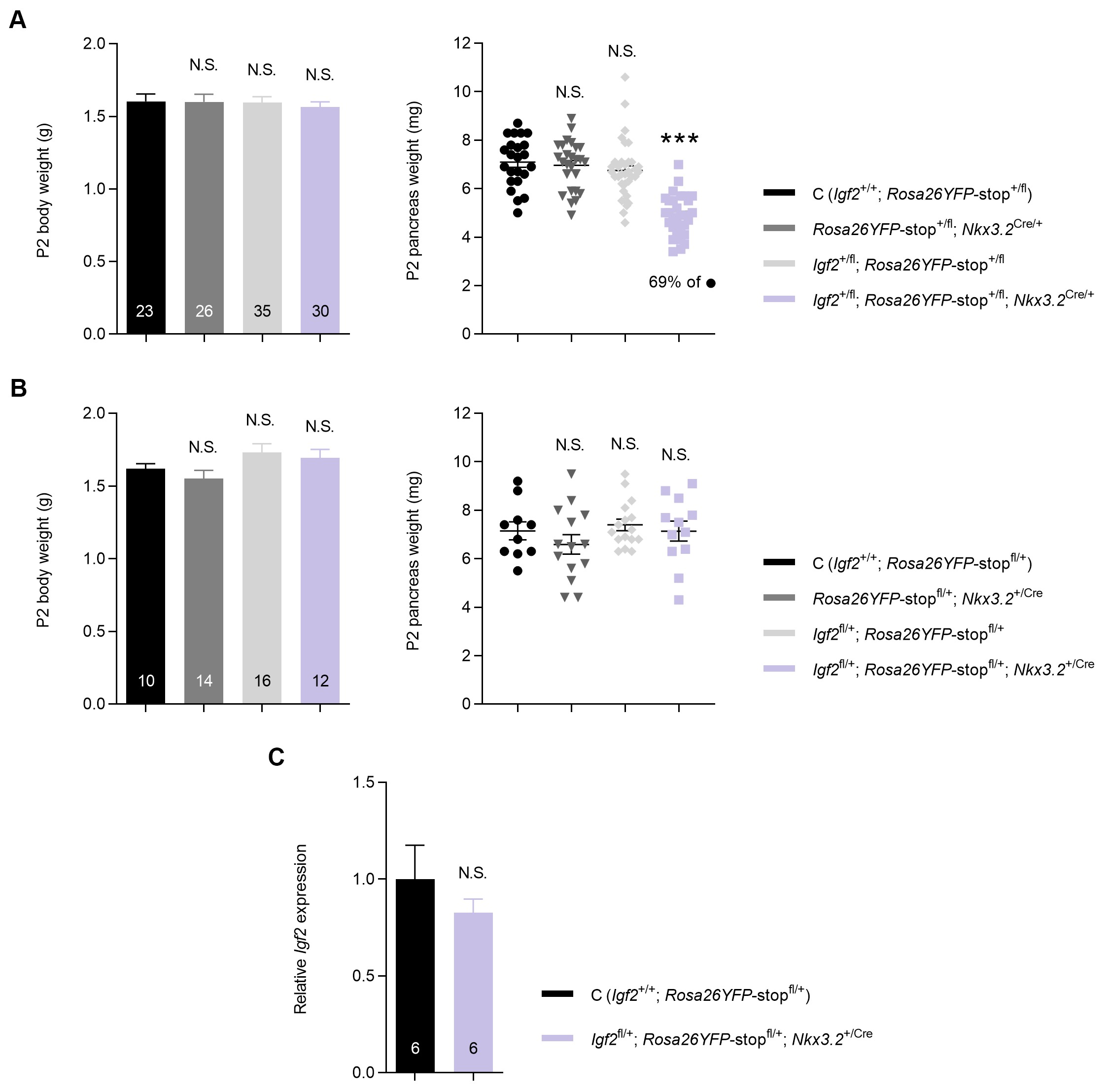

Supplement: S6 Fig — (A) Body and pancreas weights in offspring obtained from a cross between heterozygous Nkx3.2-Cre females and heterozygous Igf2 floxed males. (B) Body and pancreas weights in offspring obtained from a cross between heterozygous Igf2 floxed females and heterozygous Nkx3.2-Cre males. (C) Igf2 mRNA levels measured by qRT-PCR in pancreases with a deletion of the maternal Igf2 allele in the mesenchyme. Data is normalized to Ppia and shown relative to average Igf2 levels in controls (C–Igf2+/+; Rosa26YFP-stopfl/+), set to 1. Data is shown as averages or individual values; error bars represent SEM. Numbers shown indicate numbers of animals for each genotype. Data was analysed using one-way ANOVA with Dunnett’s multiple comparison test against the control group for panels (A) and (B) and by unpaired Student’s t test in panel (C); N.S.–non-significant; *** p<0.001. (TIF) [file pgen.1009069.s009.tif]

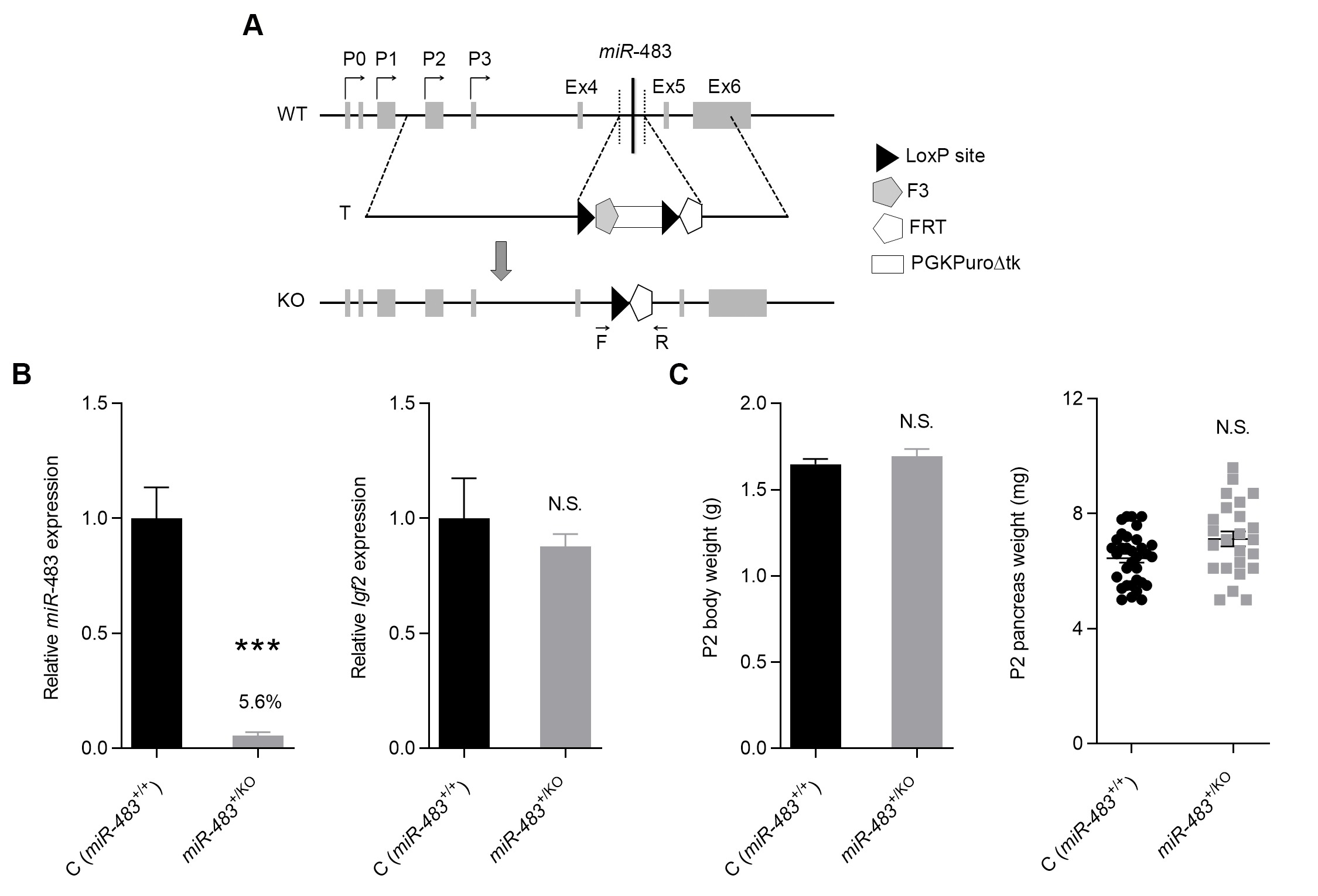

Supplement: S7 Fig — (A) Gene targeting strategy to delete the intronic Igf2 miR-483. In brief, a targeting vector was used to replace the miR-483 sequence in intron 4 of the Igf2 gene with a PGKPuroΔtk selection cassette in ES cells. Removal of the selection marker was achieved by Cre-recombination between loxP sites. Germline transmitting miR-483 KO chimeric mice were generated by ES cell injection into blastocysts (Sekita, Prosser, Zvetkova et al., manuscript in preparation). (WT–wild-type allele, T–targeted allele, KO–knock-out allele, P0 –P3 are alternative Igf2 promoters, Ex4 –Ex6 are the Igf2 coding exons, F and R indicate the position of the forward and reverse primers used for PCR genotyping). (B) miR-483 and Igf2 expression levels measured by qRT-PCR in postnatal day 2 (P2) whole pancreas from offspring of heterozygous miR-483 KO males mated with wild-type females and shown relative (%) to littermate controls (C–miR-483+/+) set to 1. Expression data was normalized to snoR-202 and snoR-234 (for miR-483) and to Ppia (for Igf2) and is shown as average + SEM (n = 10 C and n = 10 KO). N.S.–non-significant; *** p<0.001 by unpaired Student’s t test. (C) Total body weights and pancreas weights. Data is shown as averages or individual values (n = 33 C and n = 24 KO); error bars represent SEM; N.S.–non-significant differences by two-way ANOVA using genotype and litter as factors. (TIF) [file pgen.1009069.s010.tif]

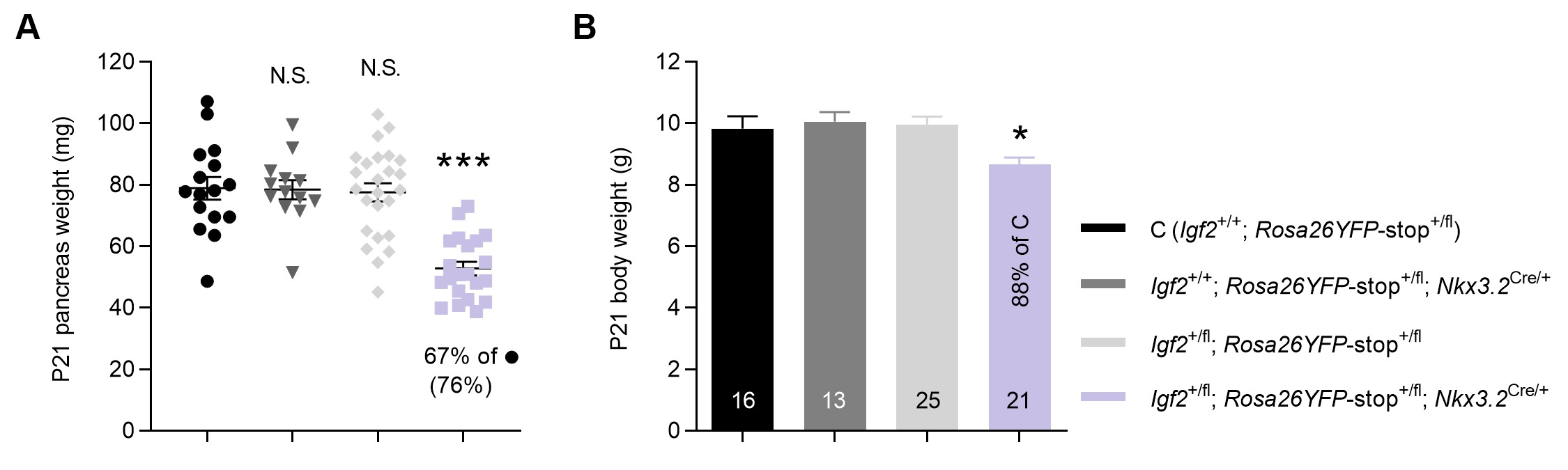

Supplement: S8 Fig — (A) Total pancreas weights and (B) body weights in offspring obtained from a cross between heterozygous Nkx3.2-Cre females and Igf2 floxed males. The value within brackets in (A) shows % pancreas weight reduction after normalization to body weight. Data is shown as individual values or averages; error bars represent SEM. Numbers of mice for each genotype are shown. Data was analysed using one-way ANOVA with Dunnett’s multiple comparison tests against the control group (C–Igf2+/+; Rosa26YFP-stop+/fl); N.S.–non-significant; * p<0.05; *** p<0.001. (TIF) [file pgen.1009069.s011.tif]
