# Supplementary material for: Mesenchyme-derived IGF2 is a major paracrine regulator of pancreatic growth and function
Source: PLoS Genet. 2020 Oct 15;16(10):e1009069. doi: 10.1371/journal.pgen.1009069 (PMC7678979; doi:10.1371/journal.pgen.1009069)
Supplement: S1 Table — (DOCX) [file pgen.1009069.s012.docx]

**S1 Table.** **Mouse strains and crosses.**

| **Paternal genotype** | **Maternal genotype** | **Offspring/embryo genotypes** | **Related to Figure** |
| --- | --- | --- | --- |
| *Igf2*^+/+^ | *Igf2*^+/+^ | *Igf2*^+/+^ | Fig 1C  Fig 4D–I |
| *Igf2*^+/fl^ | *Igf2*^+/+^ | *Igf2*^+/fl^ | S3 Fig |
|  |  | *Igf2*^+/+^ |  |
| *Igf2*^+/fl^ | *CMV*^Cre/Cre^ | *Igf2*^+/fl^; *CMV*^Cre/+^ | S4 Fig |
|  |  | *Igf2*^+/+^; *CMV*^Cre/+^ |  |
| *Igf2*^+/fl^;  *Rosa26YFP*-stop^fl/fl^ | *Nkx3.2*^+/Cre^ | *Igf2*^+/fl^; *Rosa26YFP*-stop^+/fl^; *Nkx3.2*^Cre/+^ | Fig 2F  Fig 3A–3D  Fig 4A–4C, Fig 5F  S5A Fig, S7 Fig |
|  |  | I*gf2*^+/+^; *Rosa26YFP*-stop^+/fl^; *Nkx3.2*^Cre/+^ |  |
|  |  | *Igf2*^+/fl^; *Rosa26YFP*-stop^+/fl^ |  |
|  |  | *Igf2*^+/+^; *Rosa26YFP*-stop^+/fl^ |  |
| *Nkx3.2*^+/Cre^ | *Igf2*^+/fl^  *Rosa26YFP*-stop^fl/fl^ | *Igf2*^fl/+^; *Rosa26YFP*-stop^fl/+^; *Nkx3.2*^+/Cre^ | S5B and S5C Fig |
|  |  | I*gf2*^+/+^; *Rosa26YFP*-stop^fl/+^; *Nkx3.2*^+/Cre^ |  |
|  |  | *Igf2*^fl/+^; *Rosa26YFP*-stop^fl/+^ |  |
|  |  | *Igf2*^+/+^; *Rosa26YFP*-stop^fl/+^ |  |
| *Rosa26YFP*-stop^fl/fl^ | *Nkx3.2*^+/Cre^ | *Rosa26YFP*-stop^+/fl^; *Nkx3.2*^Cre/+^ | Fig 1A, 1B  Fig 2A–2C  S1A Fig  S2A, S2B, S2D Fig |
|  |  | *Rosa26YFP*-stop^+/fl^ |  |
| *Nkx3.2*^+/Cre^ | *H19DMD*^fl/+^; *Rosa26YFP*-stop^fl/fl^ | *H19DMD*^fl/+^; *Rosa26YFP*-stop^fl/+^; *Nkx3.2*^+/Cre^ | Fig 3E–3H |
|  |  | *H19DMD*^+/+^; *Rosa26YFP*-stop^fl/+^; *Nkx3.2*^+/Cre^ |  |
|  |  | *H19DMD*^fl/+^; *Rosa26YFP*-stop^fl/+^ |  |
|  |  | *H19DMD*^+/+^; *Rosa26YFP*-stop^fl/+^ |  |
| *Nkx3.2*^+/Cre^ | *Igf2r*^fl/+^; *Rosa26YFP*-stop^fl/fl^ | *Igf2r*^fl/+^; *Rosa26YFP*-stop^fl/+^; *Nkx3.2*^+/Cre^ | Fig 3I, 3J |
|  |  | *Igf2r*^+/+^; *Rosa26YFP*-stop^fl/+^; *Nkx3.2*^+/Cre^ |  |
|  |  | *Igf2r*^fl/+^; *Rosa26YFP*-stop^fl/+^ |  |
|  |  | *Igf2r*^+/+^; *Rosa26YFP*-stop^fl/+^ |  |
| *Rosa26YFP*-stop^fl/fl^ | *RIP*^Cre/Cre^ | *Rosa26YFP*-stop^+/fl^; *RIP*^Cre/+^ | Fig 1A and 1B  S1A Fig |
| *Igf2*^fl/fl^  *Rosa26YFP*-stop^fl/fl^ | *Nkx3.2*^+/Cre^;  *Ptf1a*^+/Cre^ | *Igf2*^+/fl^; *Rosa26YFP*-stop^+/fl^; *Nkx3.2*^Cre/+^; *Ptf1a*^Cre/+^ | Fig 2F  Fig 5B–E |
|  |  | *Igf2*^+/fl^; *Rosa26YFP*-stop^+/fl^; *Nkx3.2*^Cre/+^ |  |
|  |  | *Igf2*^+/fl^; *Rosa26YFP*-stop^+/fl^; *Ptf1a*^Cre/+^ |  |
|  |  | *Igf2*^+/fl^; *Rosa26YFP*-stop^+/fl^ |  |
| *Igf2*^fl/fl^  *Rosa26YFP*-stop^fl/fl^ | *Tek*^+/Cre^ | *Igf2*^+/fl^; *Tek*^Cre/+^ | Fig 2F |
|  |  | *Igf2*^+/fl^ |  |
| *Rosa26YFP*-stop^fl/fl^ | *Ptf1a*^+/Cre^ | *Rosa26YFP*-stop^+/fl^ | Fig 1A |
|  |  | *Rosa26YFP*-stop^+/fl^; *Ptf1a*^Cre/+^ |  |
| *Igf2*^+/fl^  *Rosa26YFP*-stop^fl/fl^ | *Ptf1a*^+/Cre^ | *Igf2*^+/fl^; *Rosa26YFP*-stop^+/fl^; *Ptf1a*^Cre/+^ | S1B Fig |
|  |  | *Igf2*^+/fl^; *Rosa26YFP*-stop^+/fl^ |  |
|  |  | *Igf2*^+/+^; *Rosa26YFP*-stop^+/fl^; *Ptf1a*^Cre/+^ |  |
|  |  | *Igf2*^+/+^; *Rosa26YFP*-stop^+/fl^ |  |
| *miR-483*^+/ko^ | *miR-483*^+/+^ | *miR-483*^+/ko^ | S6 Fig |
|  |  | *miR-483*^+/+^ |  |

+ wild-type allele; fl – floxed allele; ko – knockout
