# Supplementary material for: Mesenchyme-derived IGF2 is a major paracrine regulator of pancreatic growth and function
Source: PLoS Genet. 2020 Oct 15;16(10):e1009069. doi: 10.1371/journal.pgen.1009069 (PMC7678979; doi:10.1371/journal.pgen.1009069)
Supplement: S2 Table — (DOCX) [file pgen.1009069.s013.docx]

**S2 Table.** **Primers used for genotyping by PCR.**

| Strain | Primer | Sequence (5’ to 3’) | Primer | Sequence (5’ to 3’) | Amplicon (bp) |
| --- | --- | --- | --- | --- | --- |
| *Igf2*^+/fl^ | F | TTACAGTTCAAAGCCACCACG | RW  RD | GCCAAAGAGATGAGAAGCACC  GCCAAACACAGTAAAAAGAAATGC | WT: 324  fl: 449  del: 384 |
| *H19DMD*^fl/+^ | F | CAGGCCTGTCCTCACCTGAAC | R | GCCAGCTTGCCTTGGCAACCCCTT | WT: 387  fl: 520 |
| *Igf2r*^fl/+^ | F | CCTTCCCTCCAGGCCGTTAC | R | GGTGAGGTCTCCATCTGAGTACC | WT: 225  fl: 259 |
| *miR-483*^+/ko^ | F | TACCTGCCTGTGAACTGCTCTG | R | ATCTGGTGCCTCCTGTCTGGTA | WT: 440  KO: 457 |
| *Nkx3.2*-Cre | F | CCAGGCGATCCTCAACAAGAAGAGGG | R-Cre  R-WT | CTCGTTCTCTTCGCTCAGGGCTGAG  GCCGCATAACCAGTGAAACAGCATTGC | Cre: 420  WT: 264 |
| *Ptf1a*-Cre | F | ACCTTTGCCTTCCCCAAC | R-WT  R-Cre | TGGTGGCTGAGGAACTCTAC  TGAGTGAACGAACCTGGTCG | WT: 286  Cre: 590 |
| *RIP*-Cre | F-WT  F-Cre | ATGTCTCCAATCCTTGAACACTG  CGAGTGATGAGGTTCGCAAG | R-WT  R-Cre | GCAGTGGGAGAAATCAGAACC  TGAGTGAACGAACCTGGTCG | WT: 254  Cre: 390 |
| *Rosa26YFP-*stop^+/fl^ | F | TGTTATCAGTAAGGGAGCT | R-WT  R-fl | CACACCAGGTTAGCCTTTA  AAGACCGCGAAGAGTTTGT | WT: 239  fl: 301 |
| *Tek*-Cre | F | TGTAAACAAGAGCGAGTGGA | R-WT  R-Cre | AGAGAATGGCGAGAAGTCAC  TGAGTGAACGAACCTGGTCG | WT: 240  Cre: 610 |
| *Sry* | F-WT  F-M | ATGTCTCCAATCCTTGAACACTG  CCCAGCATGCAAAATACAGA | R-WT  R-M | GCAGTGGGAGAAATCAGAACC  TCAACAGGCTGCCAATAAAA | WT: 254  M: 350 |
| *CMV*-Cre | F-WT  F-Cre | ATGTCTCCAATCCTTGAACACTG | R-WT  R-Cre | GCAGTGGGAGAAATCAGAACC | WT: 254  Cre: |

+ wild-type allele; fl – floxed allele; ko – knockout
