# Supplementary material for: Mesenchyme-derived IGF2 is a major paracrine regulator of pancreatic growth and function
Source: PLoS Genet. 2020 Oct 15;16(10):e1009069. doi: 10.1371/journal.pgen.1009069 (PMC7678979; doi:10.1371/journal.pgen.1009069)
Supplement: S3 Table — (DOCX) [file pgen.1009069.s014.docx]

**S3 Table.** **Conditions used for pancreas immunostaining.**

| Staining | Antigen retrieval | Blocking | Primary antibody | Secondary antibody |
| --- | --- | --- | --- | --- |
| Amylase | Autoclaving for 15 min at 121°C in citric acid buffer (10 mM citric acid, pH 6.0, 0.05% tween) | 5% Donkey serum (Sigma) | Rabbit polyclonal to amylase (1:100, Sigma A8273) overnight at 4°C | Donkey anti-rabbit (1:200, Jackson labs, Alexa Fluor 594) 1 hour at room temperature |
| YFP | Autoclaving for 15 min at 121°C in citric acid buffer (10 mM citric acid, pH 6.0, 0.05% tween) | 5% Donkey serum (Sigma) | Goat polyclonal to GFP (1:200, Abcam ab6673) overnight at 4°C | Donkey anti-goat (1:200, Jackson labs, Alexa Fluor 488) 1 hour at room temperature |
| IGF2 | Digestion with 1% pronase (Protease from Streptomyces griseus, Sigma Aldrich P6911) in 1xPBS for 10 min at 37°C | 15% Donkey serum (Sigma) | Goat anti-human IGF2 (1:50, R&D systems AF-292) overnight at 4°C | Donkey anti-goat (1:200, Jackson labs, Alexa Fluor 488) 1 hour at room temperature |
| Cytokeratin | Autoclaving for 15 min at 121°C in citric acid buffer (10 mM citric acid, pH 6.0, 0.05% tween) | 15% Donkey serum (Sigma) | Rabbit anti-cytokeratin (1:500, Dako) overnight at 4°C | Donkey anti-rabbit (1:200, Jackson labs, Alexa Fluor 594) 1 hour at room temperature |
| CD31 | Autoclaving for 15 min at 121°C in citric acid buffer (10 mM citric acid, pH 6.0, 0.05% tween) | 15% Donkey serum (Sigma) | Rabbit anti-CD31 (1:50, Abcam ab28364) overnight at 4°C | Donkey anti-rabbit (1:200, Jackson labs, Alexa Fluor 594) 1 hour at room temperature |
| Insulin | Not needed | 3% H2O2 30 min at room temperature, then rabbit serum (Dako) | Polyclonal guinea-pig anti-swine insulin antibody, (1:50, Dako A0564) 90 min at room temperature | Rabbit anti-guinea pig HRP coupled (1:100, Abcam ab6771) 1 hour at room temperature, then DAB for 2 min |
