# Supplementary material for: Mesenchyme-derived IGF2 is a major paracrine regulator of pancreatic growth and function
Source: PLoS Genet. 2020 Oct 15;16(10):e1009069. doi: 10.1371/journal.pgen.1009069 (PMC7678979; doi:10.1371/journal.pgen.1009069)
Supplement: S4 Table — (DOCX) [file pgen.1009069.s015.docx]

**S4 Table.** **Primers/assays used for qRT-PCR.**

| Gene | Forward primer (5’ to 3’) | Reverse primer (5’ to 3’) | Amplicon (bp) |
| --- | --- | --- | --- |
| *Ppia* | AAGGGTTCCTCCTTTCACAGAA | GATGCCAGGACCTGTATGCTT | 146 |
| *Igf2* | AGTCCGAGAGGGACGTGTCTA | CGGACTGTCTCCAGGTGTCAT | 102 |
| *H19* | GTGTCACCAGAAGGGGAGTG | AGTGCCTCATGGGAATGGTG | 110 |
| *Igf2r* | GGAAGACACCAGAACCAGACA | TGACACTCATCCTCTGGAAGC | 103 |
| *Dlk1* | GGACGGGAAATTCTGCGAAAT | TTTCCAGAGAACCCAGGTGTG | 126 |
| *Plagl1* | CTGGCTTTCCTGCTCTCACA | AGCCCAGACAGAAAGAAGGTG | 127 |
| *Mest* | GCTCTGCACTCATGGAAGACT | AGCACAACTATCTCAGGGCTT | 107 |
| *Pnliprp1* | CCTTGGAGCCCTGAGAAGATC | CCTCAATGGTCGATGGGTCAG | 103 |
| *Try* | GATTCCTGCCAGGGTGACTC | TAGTTGCAGACCTTGGTGT | 125 |
| *Amy2* | GCAAGTGGAATGGCGAGAAG | TCGCTGATTATCATGGTTGTCC | 110 |
| *Sema5b* | GTGTTCGGCCTATCATAGC | CAGGTTGTGATGTTCTGGATCC | 139 |
| *Sfrp1* | GTCAGAGGCCATCATTGAACAT | TGTCACCGTTTTCCTTCTTCAC | 86 |
| *mmu-miR-483-3p* | TM002560 (Applied Biosystems) | | |
| *snoRNA202* | TM001232 (Applied Biosystems) | | |
| *snoRNA234* | TM001234 (Applied Biosystems) | | |
