# Supplementary material for: Mesenchyme-derived IGF2 is a major paracrine regulator of pancreatic growth and function
Source: PLoS Genet. 2020 Oct 15;16(10):e1009069. doi: 10.1371/journal.pgen.1009069 (PMC7678979; doi:10.1371/journal.pgen.1009069)
Supplement: Attachment — Submitted filename: Hammerle et al_Reply to reviewers comments.docx [file pgen.1009069.s017.docx]

We would like to thank both reviewers for their constructive evaluation of our paper. We have made every attempt to fully address these comments in the revised manuscript. This includes new experimental data on measurements of mesenchymal mass in all genetic models and beta-cell mass during pregnancy, as well the addition of biochemical and transcriptome data analyses. We believe that these revisions have resulted in a significantly improved manuscript. Point-by-point replies to the reviewers’ comments can be found below, with all changes made to the original manuscript highlighted in yellow for easier cross-reference.

**Reviewer #1:** *This study reports that parentally-imprinted genes are highly expressed in pancreatic mesenchyme-derived cells and studies the role of Igf2 in mesenchymal and epithelial pancreatic lineages. A conditional Igf2 mouse model was used to show that mesenchyme-specific Igf2 deletion results in acinar and beta-cell hypoplasia, whole-body growth restriction postnatally and maternal glucose intolerance during pregnancy. This suggests that the mesenchyme is a reservoir of IGF2 throughout life contributing to paracrine control. When Igf2 was deleted in developing pancreatic epithelium. None of the above effects were seen. Increased IGF2 levels specifically in the mesenchyme resulting from conditional Igf2 loss-of-imprinting or Igf2r deletion caused pancreatic acinar overgrowth. Ex-vivo exposure of primary acinar cells to exogenous IGF2 activated AKT and increased cell number and amylase production. Results suggest that mesenchymal Igf2 is a developmental regulator of adult pancreas size and function*.

*The data is original and the use of transgenic strains innovative to tease out the contributions of mesenchymal Igf2.*

We thank the reviewer for appreciating the originality and the amount of work presented in this study.

*However, the overall effects of the targeted deletion are quite modest both in beta cell mass with no change in glucose intolerance outside of pregnancy. This probably stems from the fact that IGF2 derives from other cell compartments in the islets also and well as the blood.*

The value of the current manuscript lies on the demonstration that, in spite of the abundance of IGF2 derived from other cellular sources, including systemically, it can be shown, by multiple lines of evidence, that paracrine IGF2 actions from the ‘stromal’ cells (i.e. mesenchyme-derived cells) of the pancreas lead to significant effects on pancreas size, in particular acinar growth. Remarkably, an effect on whole body post-natal growth is also observed from around weaning and onwards. To our knowledge, this work provides the first evidence for a role of IGF2 in postnatal growth, which is programmed in fetal life (and from a specific cell type within an organ).

As the reviewer rightly points out, the effect of the mesenchyme mutation on the beta-cell is modest. We also concur that this may have to do, in part, with other redundant paracrine sources (in particular endothelial cells), and systemically. However, removing IGF2 from circulation without affecting the growth of other organs is at the moment impossible to achieve (many other organs contribute to IGF2 levels in circulation, including endothelial cells as recently shown by us – Sandovici *et al.* doi: https://doi.org/10.1101/520536; also, the liver is not the major source for circulating IGF2, unlike for IGF1 – Sandovici *et al.* unpublished).

*The paper is something of a data dump with huge swathes of material that are quite peripheral to the central hypothesis. While these data represent a huge amount of work they tend to complicate and detract from the main message of the paper. I think it could be shortened considerably without losing the key experiments.*

These comments made us think more deeply about how to streamline the paper, for example by removing data that is more peripheral to the central hypothesis. We contemplated focusing the paper on the stronger exocrine aspects only (*i.e.* impact on acinar growth). However, after a long debate, we think that it is important to portray the paper along the lines that it affects pancreas growth and function. This means including the subtle/modest/negative effects. Because of the pertinent comments made by the reviewer, we have now clarified that a full phenotype might be masked by paracrine/endocrine redundancy (lines 377-381).

We also think it is important to show the data that *Igf2* and a number of other imprinted genes are highly enriched in mesenchyme compared to the epithelium. This is of interest to the imprinting community. Little attention has been paid to the action of imprinted genes in mesenchyme-derived cells, in spite of old reports that point towards particularly important roles for genomic imprinting in mesoderm. Likewise, we feel it is important to highlight the enrichment of other novel pathways (such as semaphorin-plexin and insulin-like growth factor receptor signalling) that may play key roles in mesenchyme function of the pancreas. These findings, although peripheral to the main message of our study, are in our view important novelty points that should be included, and that merit featuring in the main figures.

*Below are a number of points that could be addressed:*

*The latter part of the Introduction would be better placed in the Discussion as this sums up the results*.

As suggested, we removed the last part of the introduction that sums up the main findings. This has shortened the manuscript by approximately 150 words (as well as avoiding repetition with interpretation of main findings mentioned in the discussion).

*It is stated that Nkx3.2 expression occurs in the mesenchyme of the developing pancreas, stomach and gut, as well as in the forming somites, but not in the endoderm-derived cells of these organs. Is epithelial to mesenchymal transformation a feature of migration of the endocrine progenitors out of the embryonic pancreatic ductal tree to form the dispersed islets at all? This might complicate the transcription factor identification of mesenchyme vs. endoderm cell types.*

This is a good point and we thank the reviewer for this comment. Current literature seems to exclude EMT (epithelial to mesenchymal transition) as a potential issue for our study (and data interpretation). Accordingly, a recent study demonstrated that aberrant EMT initiation is normally suppressed via NF-kB signalling during pancreas development and this suppression allows differentiation of mouse progenitor epithelia into key pancreatic lineages: acinar, ductal, and endocrine (Braitsch CM *et al.* 2019, PMID: 31323030). Additionally, *Nkx3.2*-Cre, used as tool to conditionally delete or overexpress *Igf2*, is active in mesenchyme around E9.5-E12.5, with no evidence for reactivation during later stages of development (Verzi MP *et al.* 2009, PMID: 19208343; Landsman L *et al.* 2011, PMID: 21909240).

*Figure 2B. I am not sure how these values might be interpreted. Igf1 is also highly expressed in mesenchyme and given its higher affinity for the Igf1R might be more biologically important. Also, there are numerous Igfbps expressed such that the levels of free Igf2 available for receptor signaling may be much lower than the total Igf2.*

Fig. 2B makes the point that *Igf2* and other genes related to IGF signalling are significantly enriched in the mesenchyme compared to non-mesenchyme cells. *Igf2* is the highest expressed gene at mRNA level within the mesenchyme. We agree with reviewer’s comment that *Igf1*, which is also highly expressed in mesenchyme (although its levels are one order of magnitude lower than those of *Igf2*) may play an important role in controlling pancreatic growth. However, our results for conditional *Igf2* deletion in the pancreatic mesenchyme clearly show that IGF2 actions are, at least in part, non-redundant with those of IGF1. We hope to address the role of mesenchymal *Igf1* in pancreas development in the future.

*There is no comparison given for the ectoderm compartment so it is difficult to make comparisons.*

Apologies if we have mis-interpreted this comment, but Fig. 2B actually shows levels of expression in both mesenchyme-derived cells and non-mesenchymal cells, thus allowing for a direct comparison between the two cell lineages.

*For Figure 2A how would be pathway analysis differ for any other developing tissue compartment in a rapidly growing animal? How is the pancreatic mesenchymal tissue any different from liver or kidney or lung for instance? The same could be asked for Figure 2C.*

We agree that pathway analyses in any rapidly growing organ are likely to highlight developmental processes and key signalling pathways. This is a good point, which led us to perform similar pathway analyses for the non-mesenchymal fraction. These new results, shown in the graph below (Fig. 1), highlight several processes that are specific to the non-mesenchymal compartment (i.e. enrichment of genes related to digestion, endocrine pancreas development etc.). Please note that none of the GO terms listed below (enriched in genes with higher expression levels in non-mesenchymal cells) are common with those identified in the mesenchyme fraction (in Fig. 2A), in spite of these analyses being performed at the same time point – *i.e.* of a rapidly growing animal. This graph has been added as a new panel in supplementary figure S2 (S2C Fig), which is briefly presented in results (lines 172-176). Additionally, the related data is found in the revised version of S1 Data. As a last point, we do not have mesenchyme RNA-seq datasets at P2 from other organs, and are unaware of other studies done at the same time-point, to address the second question.

Fig. 1. Top scoring biological processes containing genes enriched in the pancreatic non-mesenchyme cells as identified by DAVID functional annotation. The dotted line in panel corresponds to a p value of 0.05.

*Interpretation of a reduced pancreatic weight and beta cell mass in the mesenchymal Igf2 ko mice is solely in terms of the mitogenic effects of IGF2. However, in the developing neonatal pancreas IGF2 is anti-apoptotic and can equally influence cell number by this mechanism.*

We thank the reviewer for this comment and apologise for the omission. We added the missing information and references about the anti-apoptotic roles of IGF2 in the introduction (line 89). The pro-survival actions of IGF2 in the neonatal pancreas are also mentioned in discussion (lines 410-411).

*Within a paracrine environment of the P2 pancreas it is clear that the epithelial cells and endothelial cells are the major relative contributors to IGF2 content as opposed to mesenchyme (Fig 2F).*

We apologise if the reviewer was led by us in mis-understanding the data shown in Fig. 2F. We have now made it clearer in the figure that this relates to measurements of **residual *Igf2* mRNA levels after deletion in** **the different cell types**, and clarified in figure legend that ‘Residual levels of *Igf2* mRNA in each mutant are indicated as percentage values of control levels set to 100% (% C)’. We have also modified the wording in the text. Instead of relating the levels to % control levels, which although correct we admit it can indeed be misleading, we now unequivocally state that the mesenchyme contributes with ~75-85% to the overall *Igf2* mRNA levels, while endothelial cells contribute with approximately 20-25%, and acinar/ductal cells with less than 1%.

The conclusion remains, as stated in the text, that the mesenchyme is the major relative contributor to overall pancreatic IGF2 levels at P2 and throughout pancreas development. Consistent with this finding it is only when IGF2 is removed from the mesenchyme specifically that we see a phenotype in pancreas size and function.

*The beta cells will not differentiate between IGF2 derived from any source, including that in the circulation in the young mouse. Again, not sure how this data reinforces the hypothesis.*

As demonstrated by the epithelial deletion, which does not have an effect on pancreas size, we suggest that there is little evidence for IGF2 paracrine effects arising from those epithelial cell types. We believe that this lack of an effect is related with the small levels of IGF2 expression in epithelium compared to those emanating from the mesenchyme. We agree with the reviewer that beta cells would not be able to differentiate between IGF2 derived from any particular source, but our data supports the hypothesis that the paracrine stimulus is mainly arriving from the mesenchyme and/or endothelial cells, and not from the neighbouring epithelium. In relation to the specific question on the effect of circulating IGF2, the effects we are seeing are independent of circulating levels as the global levels of IGF2 are not significantly changed in the blood of P2 neonates (in either the deletion or loss-of-imprinting overexpression model) (Fig. 2). These results agree with the normal weight of mutant pups at P2. Moreover, we would like to point out that paracrine effects of IGF2 have been recently found in several other organs such as the heart and brain, despite the fact that global levels of IGF2 in circulation remain largely normal (Shen H. *et al.*, 2015, PMID: 25560321; Ferrón SR *et al.* 2015, PMID: 26369386), with both of these studies using our ‘pre-publication’ conditional *Igf2* allele, whose full generation details we are now incorporating in this manuscript.

Fig. 2. Circulating IGF2 levels measured by ELISA in P2 mice. N.S. – non-significant using one-way ANOVA with Dunnett’s multiple comparison tests.

*In Fig 3 the reduction or over-expression strategies for mesenchymal Igf2 expression have clearly worked, but how about the actual numbers of mesenchymal cells in each? If the mesenchymal IGF2 is a growth factor for the mesenchymal cells the reduction or increase in pancreas weight may reflect changes on the mesenchymal cell number. The reduction in beta cell mass in Fig 3D is modest, and no such data is shown for the mesenchymal Igf2 over-expression model.*

We have addressed this criticism by adding into the results section the stereological measurements of the mesenchymal cell mass for all genetic models, and beta-cell mass for the overexpressor models (see Fig. 3 below for the complete set). In the revised manuscript Fig 3 is replaced by Figs 3 and 4 and the interpretations of these results is added to the discussion (lines 344-351 and 420-424).

As already shown in the original submission, the acinar compartment provides a clear picture, *i.e.* being reduced in mesenchymal *Igf2* KO and increased in the two models of mesenchymal IGF2 over-expression. Regarding the mesenchyme mass measurements, we found no evidence for reduced mesenchymal mass when IGF2 was deleted from mesenchyme cells, or evidence for an increase in mesenchymal mass when IGF2 was overexpressed in the IGF2R mesenchyme-specific model. These findings have 2 important implications: 1) that the effects seen in the acinar compartment, in particular, are not directly caused by loss or increased mass of the mesenchyme; 2- it further suggests that the main function of IGF2 in mesenchyme is to act as a developmental reservoir of this peptide for paracrine signalling (*i.e.* rapid secretion to extra-cellular milieu).

Although the analysis of these 2 models suggests that there is no evidence for an autocrine function for IGF2 in the mesenchyme, a full appraisal of this issue will require further studies during fetal and post-natal development. This more careful approach is also required as the loss-of-imprinting model H19/DMD is associated with an increased mesenchymal mass. Regarding the beta-cell mass, although reduced in the mesenchymal *Igf2* KO and with a trend for being increased in the mesenchymal *Igf2r* KO, it shows no significant change in the mesenchymal *H19DMD* KO. As we mention in the discussion (lines 420-424), both models of mesenchymal IGF2 over-expression have some limitations (*e.g.* reduced *H19* and *miR-675* expression in the mesenchymal *H19DMD* KO and altered binding for mannose 6-phosphate in the mesenchymal *Igf2r* KO that may explain some of these discrepancies).

Fig. 3. Measurements of acinar, beta cell and mesenchyme mass compartments using pancreas stereology in mutants (Mut), compared to littermate controls (C). For all graphs, data is shown as individual values and averages ± SD; N.S. – non-significant, * p<0.05, ** p<0.01 by unpaired Student’s *t* tests.

*Figure 4 is not surprising in that if you deleted the expression of any growth factor from a tissue compartment you would see changes in the expression of other classes of genes. What is this specifically indicating about mesenchymal Igf2? Deletion of the mesenchymal Igf2 appears to be invoking the expression of immune system and chemokine genes. Therefore, is the overall level of inflammation altered in these animals, which could explain later phenotypic changes?*

We would like to clarify that the RNA-seq data was obtained in mesenchymal cells (in which *Igf2* deletion takes place), and in the matching neighbouring non-mesenchymal cells (without deletion of *Igf2*). This latter RNA-Seq analysis was designed to address the effects of deleting IGF2 from one compartment on the other (hence paracrine effects). We appreciate and entirely agree with the reviewer when saying that changes are expected in other genes when taking a growth factor from a tissue. What was surprising was to see very few changes in the mesenchyme itself, perhaps consistent with the stereological findings that mesenchymal mass is not altered in the *Igf2* mesenchyme specific KO (please see new data and comments above). In line with a major paracrine effect, the expression changes are more abundant and severe in the non-mesenchymal cells. Additionally, as the reviewer points out, the non-mesenchymal cells show several expression signatures, including increased expression of chemokines and immune cells. We have not assessed whether the mutants exhibit global inflammation or a local pancreatitis, which we hope to address in the future, as part of investigations to find out why these mice are growth restricted postnatally. However, the potential role of inflammation in the phenotype observed is discussed (lines 368-373).

*If the mesenchymal Igf2 ko mice were stressed postnatally through a high fat diet would they become diabetic due to underlying beta cell metabolic stress? This might also underlie the impaired glucose tolerance seen in pregnancy. The pregnancy experiments could be extended as an adaptive 2-3 fold increase in beta cell mass normally occurs in mice by gestational day 19. Was this impaired in the Igf2 ko mice?*

Prompted by this pertinent comment by Reviewer 1 and a similar comment by Reviewer 2, we investigated if beta-cell mass is altered in the pregnant mutant females compared to pregnant controls. Our results show that pregnant females lacking mesenchymal IGF2 have a reduced beta-cell mass, with the deficit similar to that observed in early postnatal life (~82% at P2 and ~84% in pregnant females) (see Fig. 4 shown below). This data is now incorporated in the revised manuscript as Fig. 6G. Although this observation might be suggestive that beta-cell mass expansion proceeds at a normal rate in pregnant females lacking mesenchymal IGF2, further work is required in later pregnancy to confirm this preliminary finding, including the addition of a number of appropriate controls. In the revised manuscript, we only highlight these new findings as confirmation of beta-cell mass defect in post-natal adult animals (see also comments to Reviewer 2), and suggest that this might be a contributing factor to the glucose intolerance phenotype (lines 298-303 and 377-381 and updated Fig. 6). Consequently, beta-cell mass measurements were done at E15.5, to coincide with the impaired glucose tolerance time-point. Lastly, we did not expose the mesenchymal *Igf2* KO mice to high fat diet. We are planning to perform nutritional manipulations in the future, as part of investigations aimed at identifying the mechanisms of glucose homeostasis regulation in both pregnant and non-pregnant mesenchyme and double mesenchyme + beta cell IGF2 KOs (see also comments to Reviewer 2).

Fig. 4. Beta cell mass measurement in pregnant females. Beta cell mass was measured by pancreas stereology in E15 pregnant females [n = 10 controls (C) and n = 9 mutants (Mut)]. Data is shown as individual values and averages ± SD. * p < 0.05 using an unpaired Student *t* test.

**Reviewer #2:** *Review of Mesenchyme-derived IGF2 is a major paracrine regulator of pancreatic growth and function*

*This manuscript from Hammerle et al reports on the role of mesenchymal IGF2 and IGF2 signaling in the control of pancreas size. This is very compelling data set using multiple lineage specific loss-of-function models, as well as gain-of-function model to make the case the imprinted Igf2 in the mesenchyme plays a critical role.*

*This is an understudied area and the present results break important new ground.*

*The data and figures are very well presented. The approach is systematic.*

*This is an exceptional paper.*

We are very appreciative of the very positive assessment of our work.

*Minor comments/questions:*

*Does the IGF2 act solely via the IGF2R on the acinar cells, or is there a role for INSR or INSR/IGF2R hybrid receptors?*

We have made multiple attempts to identify which receptor might be responsive for the IGF signalling in acinar cells, but we were ultimately unsuccessful. This will require further extensive work, which we hope to do after securing publication of this paper and apply for further funding. The notes below explain our attempts and findings so far. We opted for not including this data in the manuscript, but would be pleased to do so, as supplementary figure, if the reviewer thinks that it adds worthwhile information.

Both IGF1R and INSR proteins are expressed in acinar cells *in vivo* and *in vitro* (Fig 5A and B). Hybrid INSR/IGF1R receptors are expressed at the cell surface as pre-formed dimers in cells that express both receptor types and are generally thought to form randomly. As such, in cells that express INSR and IGF1R in equal amounts, a ratio of 1:2:1 of INSR homodimer : hybrid receptor : IGF1R homodimer is expected. Although the limited sample size of primary acinar cell lysates precluded co-immunoprecipitation experiments to demonstrate hybrid INSR/IGF1R expression, given what is accepted about hybrid receptor formation we can assume that hybrid receptors are expressed in acinar cells. Hybrid receptors are thought to function more like IGF1R than INSR as evidenced by retaining high affinity for IGF1 and IGF2, but relatively low affinity for insulin (Benyoucef S *et al.* 2007, PMID: 17291192). RT-PCR data suggests that the INSR-B isoform (that lacks appreciable affinity for IGFs) is the predominant INSR isoform expressed in acinar cells (Fig. 5C). Thus, homodimer INSR-B are unlikely to be mediating IGF2 signalling in acinar cells. We observed phosphorylation of Ser473 of AKT, a signalling node downstream of both the INSR and IGF1R, in acinar cell cultures stimulated with increasing amounts of IGF2. **Multiple attempts were made to detect phosphorylated INSR or IGF1R in response to IGF2 stimulation, however a receptor phosphorylation response to IGF2 stimulation could not be reliably detected. Amplification of downstream signal modulators are the hallmark of signalling cascades, thus given the relatively modest phosphorylation of AKT observed, it is possible that the initiating signal arising from the receptors was too weak to be detected in the limited amount of primary cell lysate.** However, due to the high level of homology between the tyrosine kinase domains of the INSR and IGF1R, antibodies that detect phosphorylated residues within the tyrosine kinase domains are not specific for either receptor type and would not be able to distinguish activation of one receptor type over another, let alone determine hybrid vs. homodimer receptor signalling. Together with the lack of specific inhibitors to either the INSR, IGF1R, or hybrid receptors, we cannot determine which receptors mediate the actions of IGF2 on acinar cells.

Fig. 5. Expression of IGF1R and INSR *in vivo* and in primary cells cultured *ex vivo*. (A) Immunohistochemistry staining for IGF1R and INSR in pancreas at P2 (Rb IgG – negative isotype control rabbit IgG). Isl – islets, Ac – acini. Scale bars are 20 µm. (B) Western blot demonstrating higher expression of IGF1R and INSR in primary acinar cells versus mesenchyme-derived cells isolated from P2 pancreases. * indicate pro-receptors. (C) Semi-quantitative RT-PCR showing predominant expression of *Insrb* in acinar cells and *Insra* in mesenchyme-derived cells. MEFs – mouse embryonic fibroblasts; RT (-) – no reverse transcriptase; NTC – no template control (no cDNA).

*Given the emerging role for insulin and insulin like growth factor signalling on acinar and duct cells in pancreatic cancer (Zhang 2019 Cell Metabolism), could the axis described here be reactivated in adults and contribute to the initiation of pancreatic cancer?*

We thank the reviewer for this comment that brings up the potential connection between IGF2 and pancreatic cancer. There is good evidence that the IGF system is involved in the development and progression of pancreatic cancer, particularly for pancreatic ductal adenocarcinomas – PDAC (reviewed by Trajkovic-Arsic M *et al.* 2013, PMID: 23493758). However, the evidence published so far is significantly stronger for IGF1 compared to IGF2. Nevertheless, according to the Human Protein Atlas (<https://www.proteinatlas.org>) the survival rate is significantly reduced (p=0.037) in pancreatic cancer patients with high IGF2 expression (n=135, survival rate at 5 years of 22%) compared to patients with low IGF2 expression (n=41, survival rate at 5 years of 45%). It is unclear whether IGF2 is reactivated within the cancer cells (epithelium) or in the surrounding interstitial tissue derived from the mesenchyme. Please note that we have removed, in the revised paper, the potential implications of this work to cancer, as we felt that the current evidence in the literature, as explained above, is perhaps not supportive enough for IGF2.

*What is the predicted IGF2 levels within the pancreas in adults and how do these compare with the in vitro studies?*

*Igf2* expression in mouse pancreas declines rapidly after post-natal day 14 (P14) – please see Fig. 1B and note the logarithmic scale. Please also note that our *in vitro* work was done using primary pancreatic cells isolated from neonatal mice (P2). *Igf2* expression in primary mesenchymal cells derived from P2 mice is high and is well maintained over 4 days of *ex vivo* culture (Ct values in whole P2 pancreas are around 17; Ct values in cultured mesenchymal cells are between 14-16). Attempts to isolate and culture mesenchymal cells from adult mice failed (there are very few mesenchymal cells at isolation and they fail to expand). In human, IGF2 remains expressed in adulthood (DeChiara *et al.* 1990, PMID: 2330056; Vu TH & Hoffman AR, 1994, PMID: 7935819; Lui JC & Baron L, 2013, PMID: 23530192). However, cell-type specific IGF2 expression during development and adulthood has not been carefully quantified in human pancreases.

*Line 383-5: “Although we provide evidence that mesenchymal IGF2 is the long-sought promotor of acinar growth, we have not established the precise timing of the actions of IGF2.” Should be changed to “a long-sought promoter” since it is likely that there is more than one.*

We made this change, as suggested.

*In Fig. 5. It would be ideal if the authors could show insulin levels. If there are pancreas sections from this time point, and analysis of beta-cell mass would greatly improve the paper and provide some mechanistic insight into the* *impaired glucose homeostasis. The authors should elaborate a little more about what they think is underlying this pregnancy-induced defect.*

As mentioned in the answer to the related comment made by Reviewer 1, we now show in the revised manuscript that pregnant mutant females have a ~15% reduction in beta-cell mass compared to the pregnant controls. The measurements in male mice at a similar age (9 weeks, data not presented in the manuscript), also show a reduction of the beta-cell mass (see Fig. 6). This data shows that the beta-cell defect persists in adult mice, similarly to the neonates.

Fig. 6. Beta-cell mass measurement in 9-weeks old males. Beta-cell mass was measured by pancreas stereology in chow-fed males [n = 7 controls (C) and n = 7 mutants (Mut)]. Data is shown as averages ± SD. * p < 0.05 using an unpaired Student *t* test.

We measured insulin levels at basal state only (*i.e.*, after six hours of fasting, prior to the start of OGTT – T0). Both glucose and insulin levels were similar between the two genotypes at T0 (see Fig. 7 below). We added these new results to the last section of the paper (Fig. 6F and lines 298-303). We totally agree that measuring the insulin response during OGTT would have been informative regarding a possible interpretation of the impaired glucose tolerance, but unfortunately we have not collected blood to allow for those measurements.

It is likely that the altered glucose homeostasis observed during pregnancy is due to a combination of both beta-cell mass deficit and beta-cell dysfunction, but this will require a systematic targeted approach, looking at the expansion of beta-cell mass, including cell proliferation parameters at 2 times during pregnancy, together with measurements of insulin during OGTT and insulin tolerance tests. Given that the phenotype is modest, we would like to also analyse in parallel double KOs with deletion of mesenchymal and beta-cell *Igf2*. We hypothesize that, in situations of metabolic stress related either to pregnancy or to high-fat diet, disrupting the autocrine IGF2 actions from the beta-cell and the paracrine actions from the mesenchyme will be required to achieve a more severe glucose intolerance phenotype. In that regard, the Thorens lab published evidence of impaired beta-cell expansion and glucose tolerance in beta-cell deficient IGF2 mice, showing that there is an important component of the IGF2 in the beta-cells that remains intact in our mesenchymal *Igf2* KO (Modi H. *et al.*, 2015, PMID: 26384384). We are hopeful of doing these experiments with further funding.

Fig. 7. Fasting glucose (left) and insulin (right) levels in 8-week old *Igf2* mesenchyme-specific deficient pregnant females (E15 of gestation) after six hours of fasting [n=16 controls (C – *Nkx3.2-Cre*; *Igf2*^+/+^) and n=15 mutants (Mut – *Nkx3.2-Cre*; *Igf2*^+/fl^)]. Data is shown as individual values and averages ± SD. N.S. – non-significant using unpaired Student *t* tests.
